# Supplementary figures and images for: Global and context-specific transcriptional consequences of oncogenic Fbw7 mutations
Source: eLife. 2022 Feb 28;11:e74338. doi: 10.7554/eLife.74338 (PMC8926403; doi:10.7554/eLife.74338)

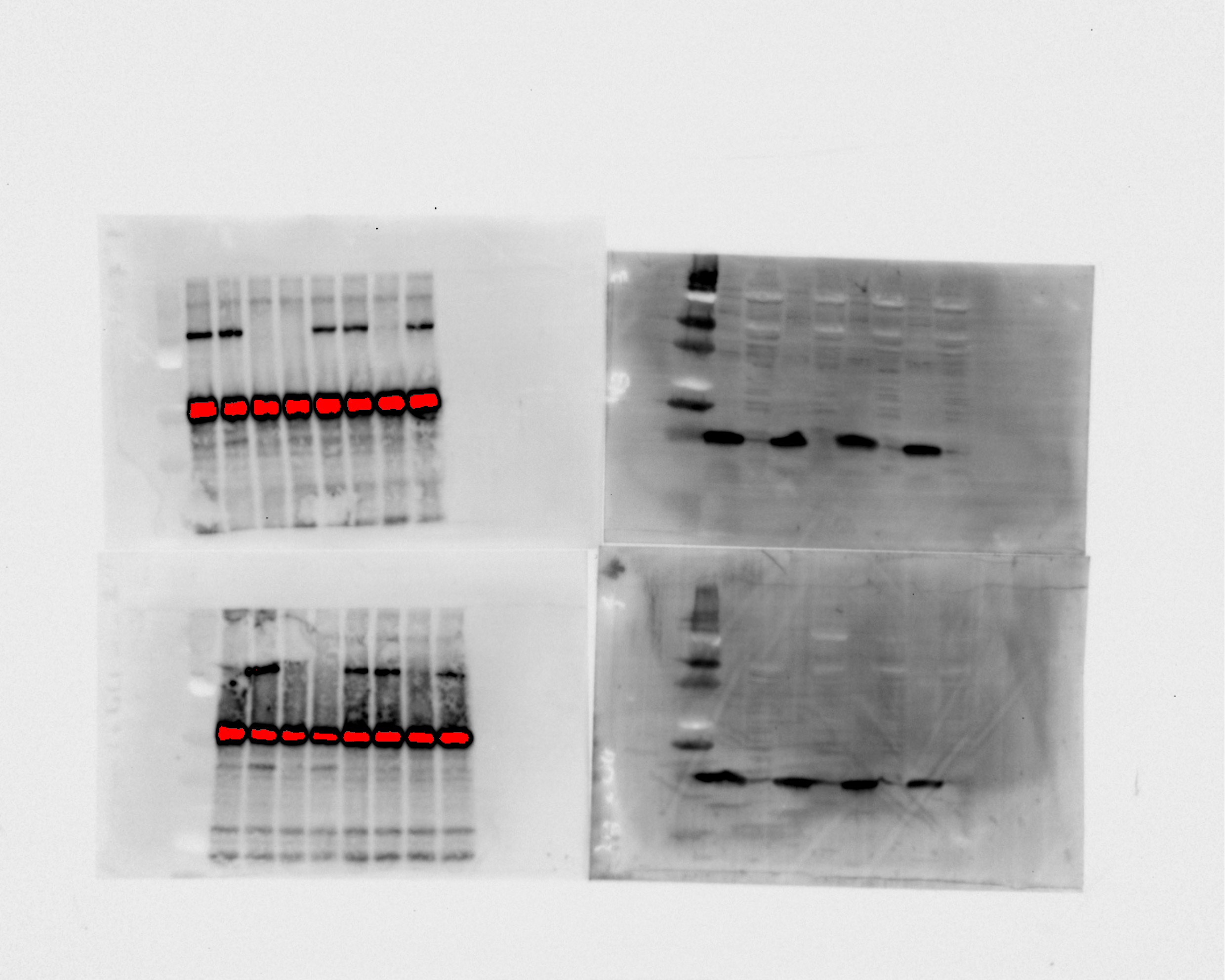

Supplement: Figure 3—source data 1. [file elife-74338-fig3-data1.zip › Figure 3 - source data 1/Figure3A_original.tif]

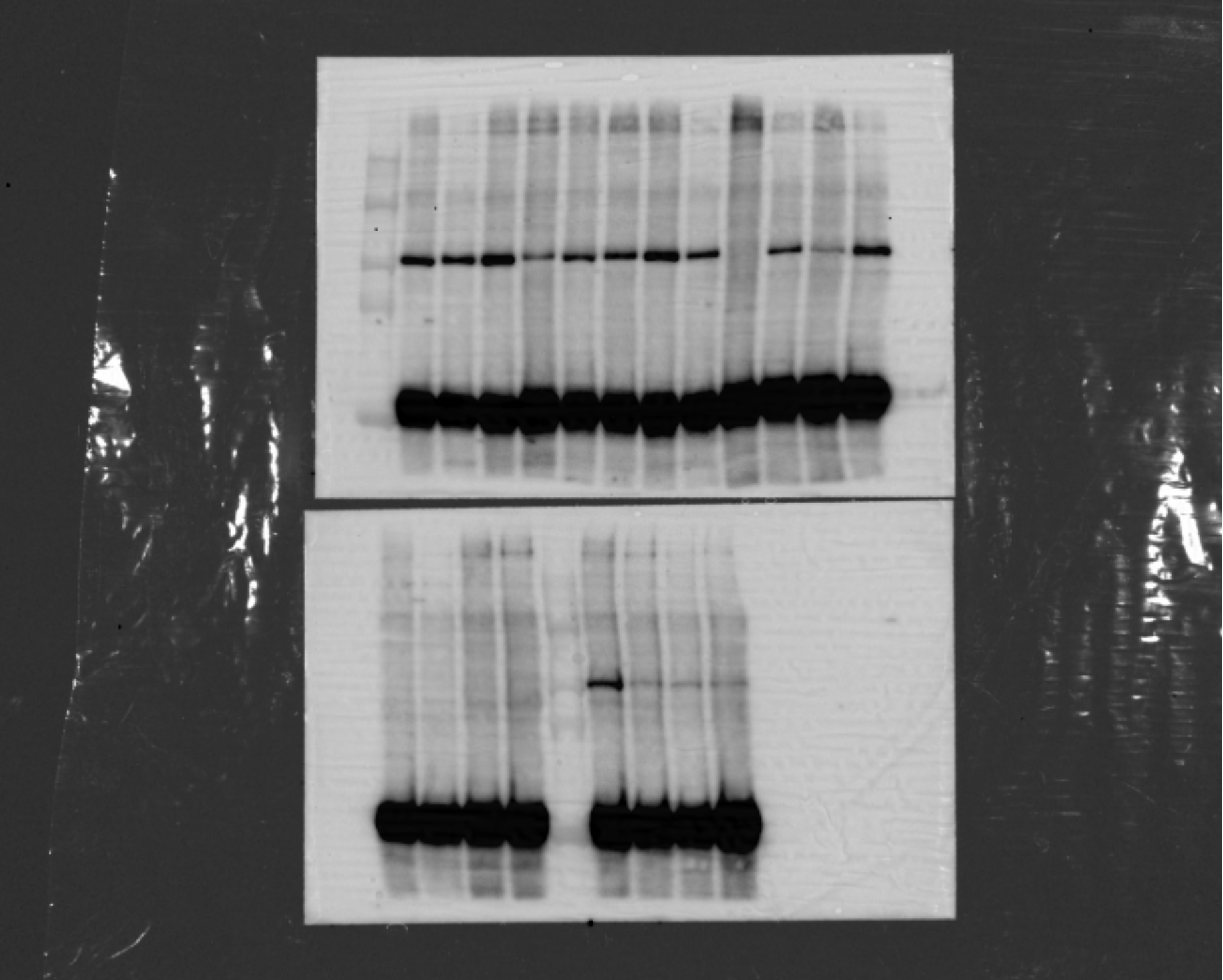

Supplement: Figure 3—source data 1. [file elife-74338-fig3-data1.zip › Figure 3 - source data 1/Figure3Sup1_original.tif]

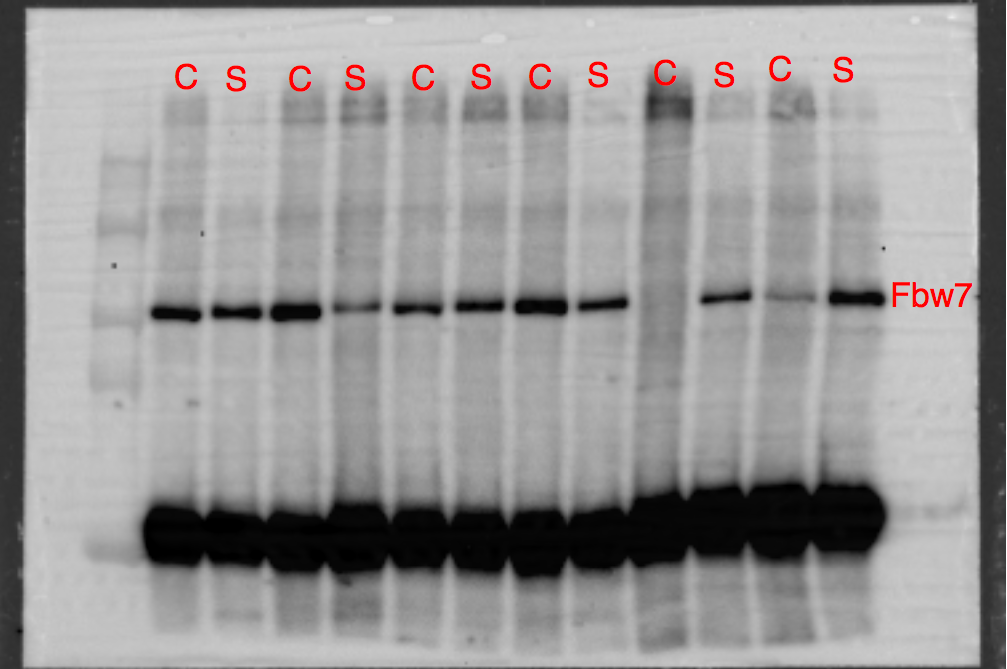

Supplement: Figure 3—source data 1. [file elife-74338-fig3-data1.zip › Figure 3 - source data 1/Figure3Sup1.tif]

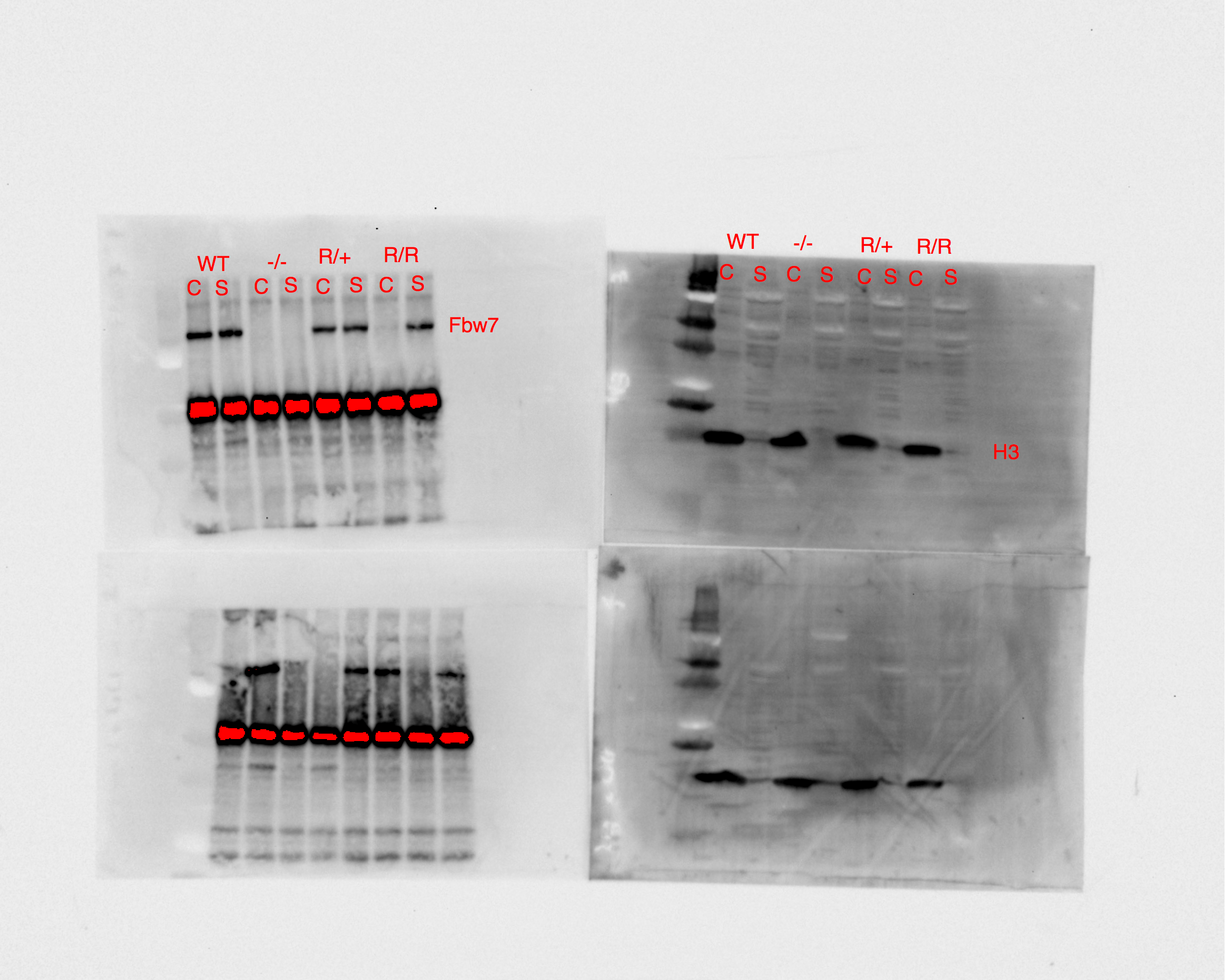

Supplement: Figure 3—source data 1. [file elife-74338-fig3-data1.zip › Figure 3 - source data 1/Figure3A.tif]

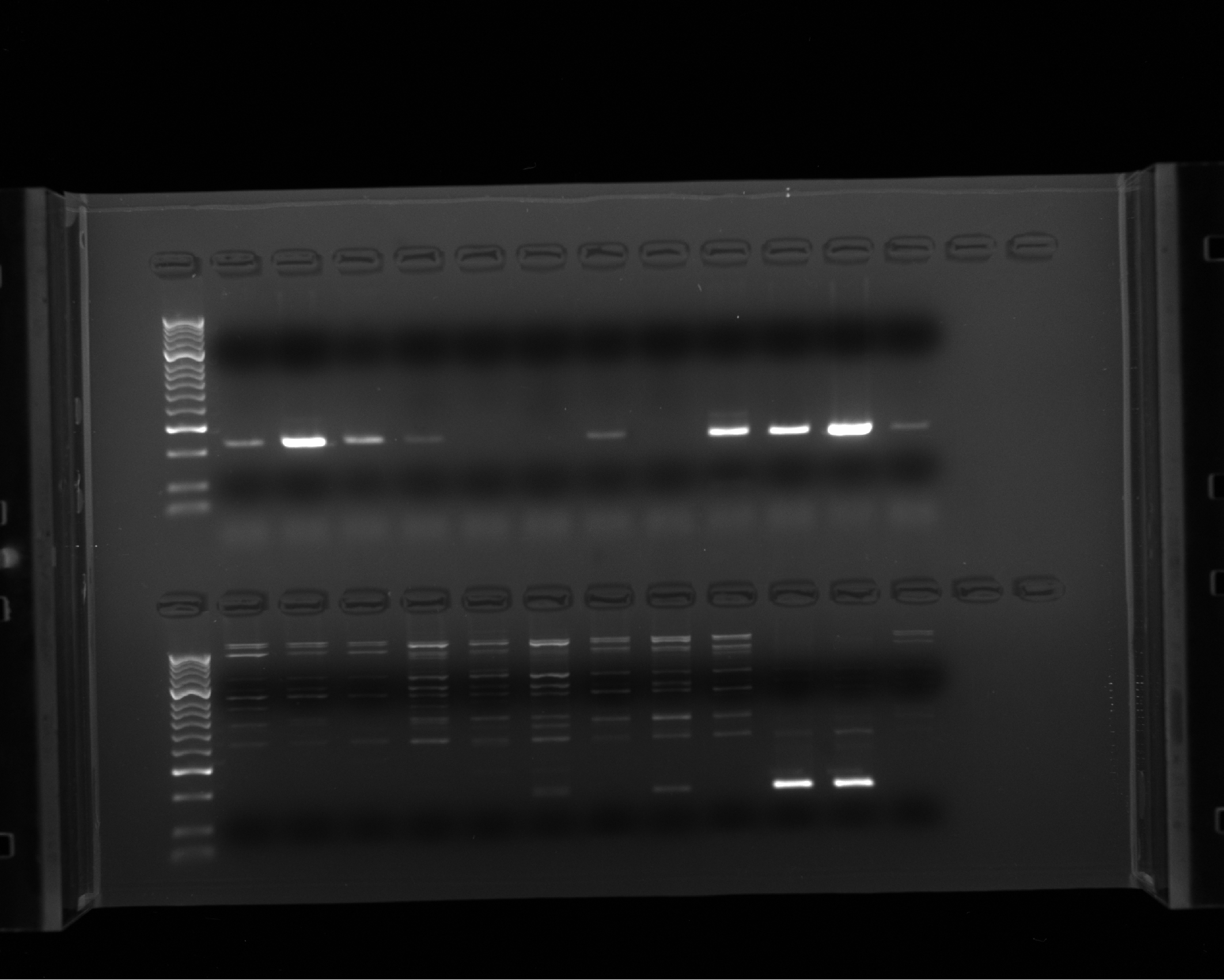

Supplement: Figure 5—source data 1. [file elife-74338-fig5-data1.zip › Figure 5- source data 1/Figure5C_original_CIITA.tif]

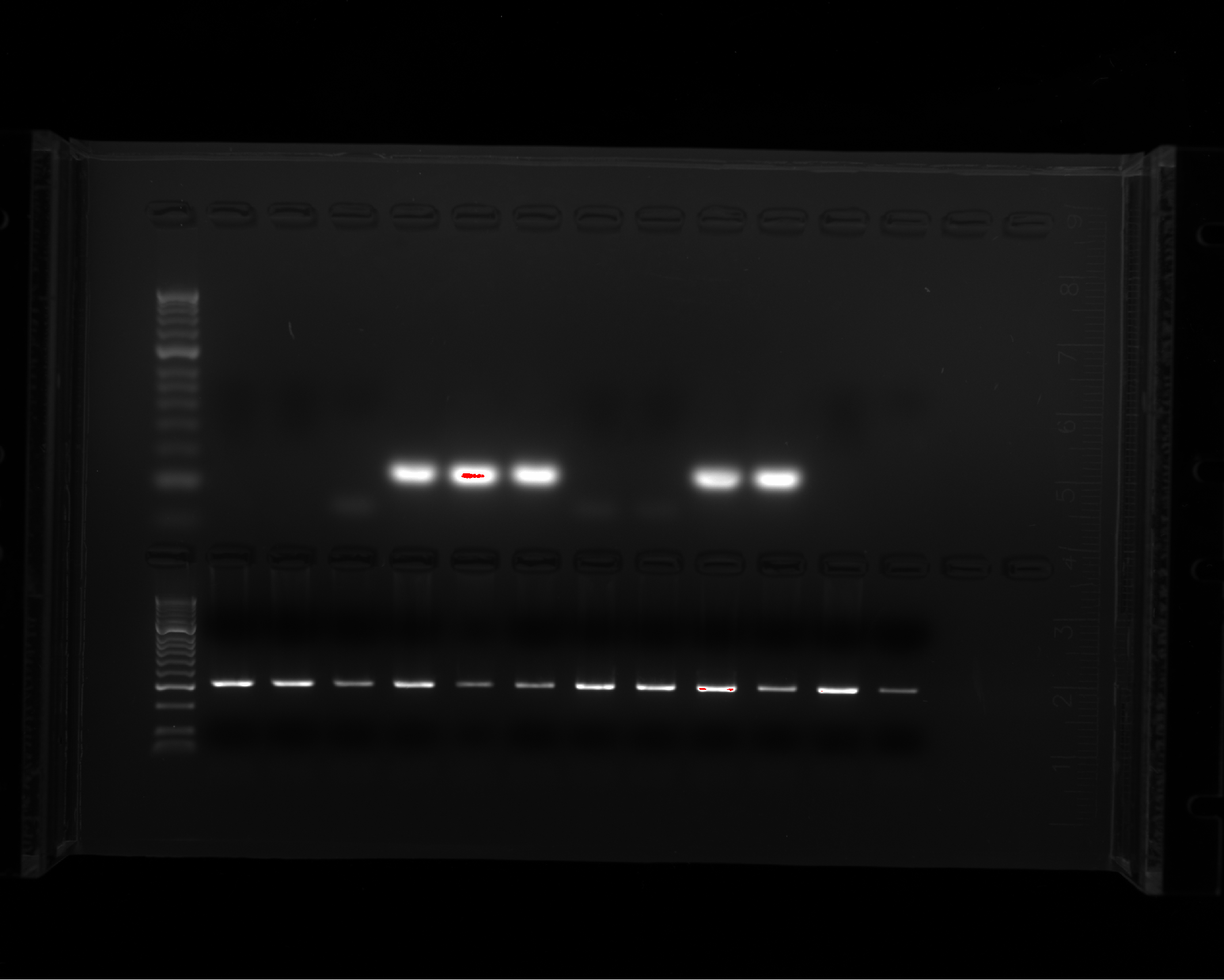

Supplement: Figure 5—source data 1. [file elife-74338-fig5-data1.zip › Figure 5- source data 1/Figure5C_orginical_GAPDH.tif]

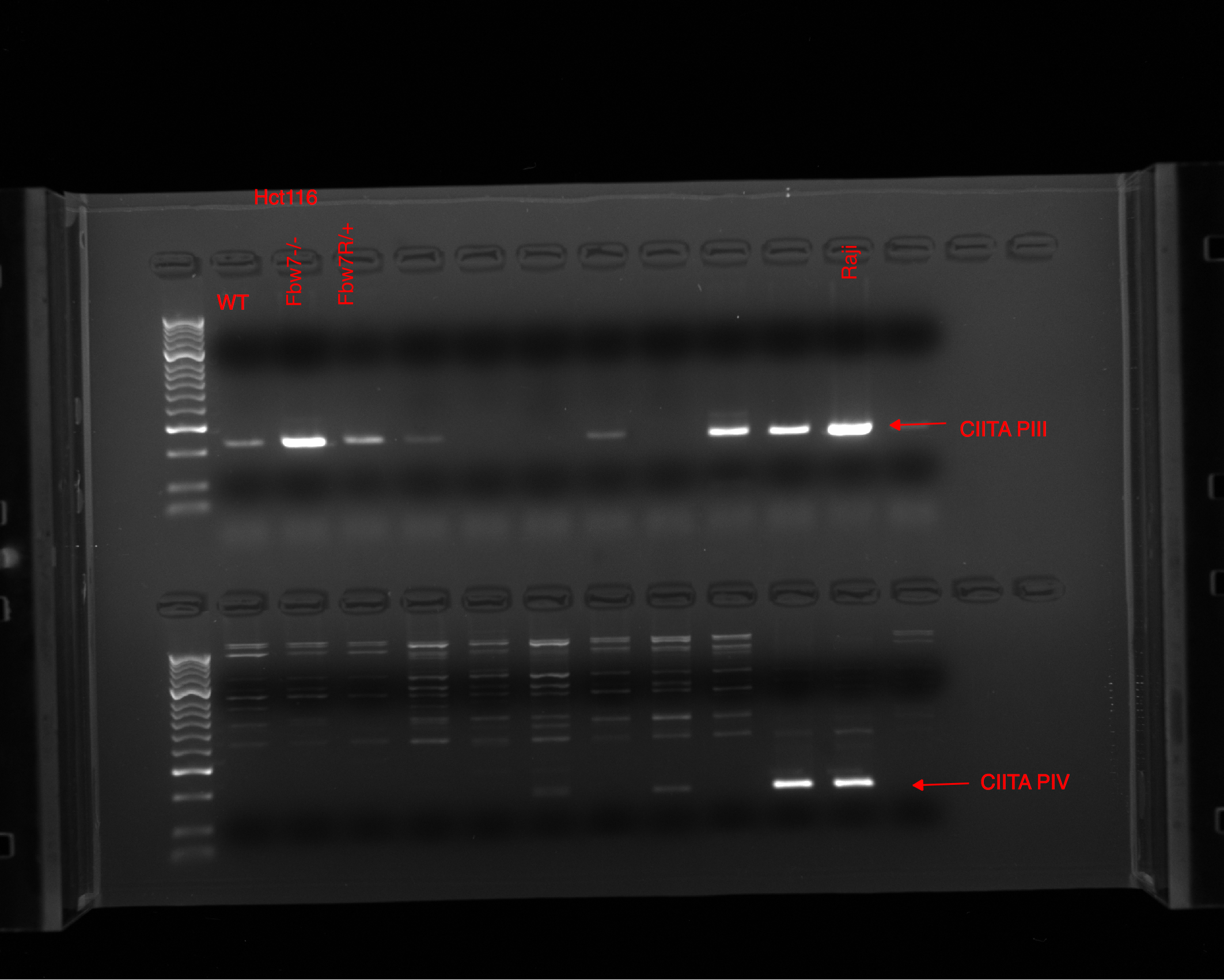

Supplement: Figure 5—source data 1. [file elife-74338-fig5-data1.zip › Figure 5- source data 1/Figure5C_labeled_CIITA.tif]

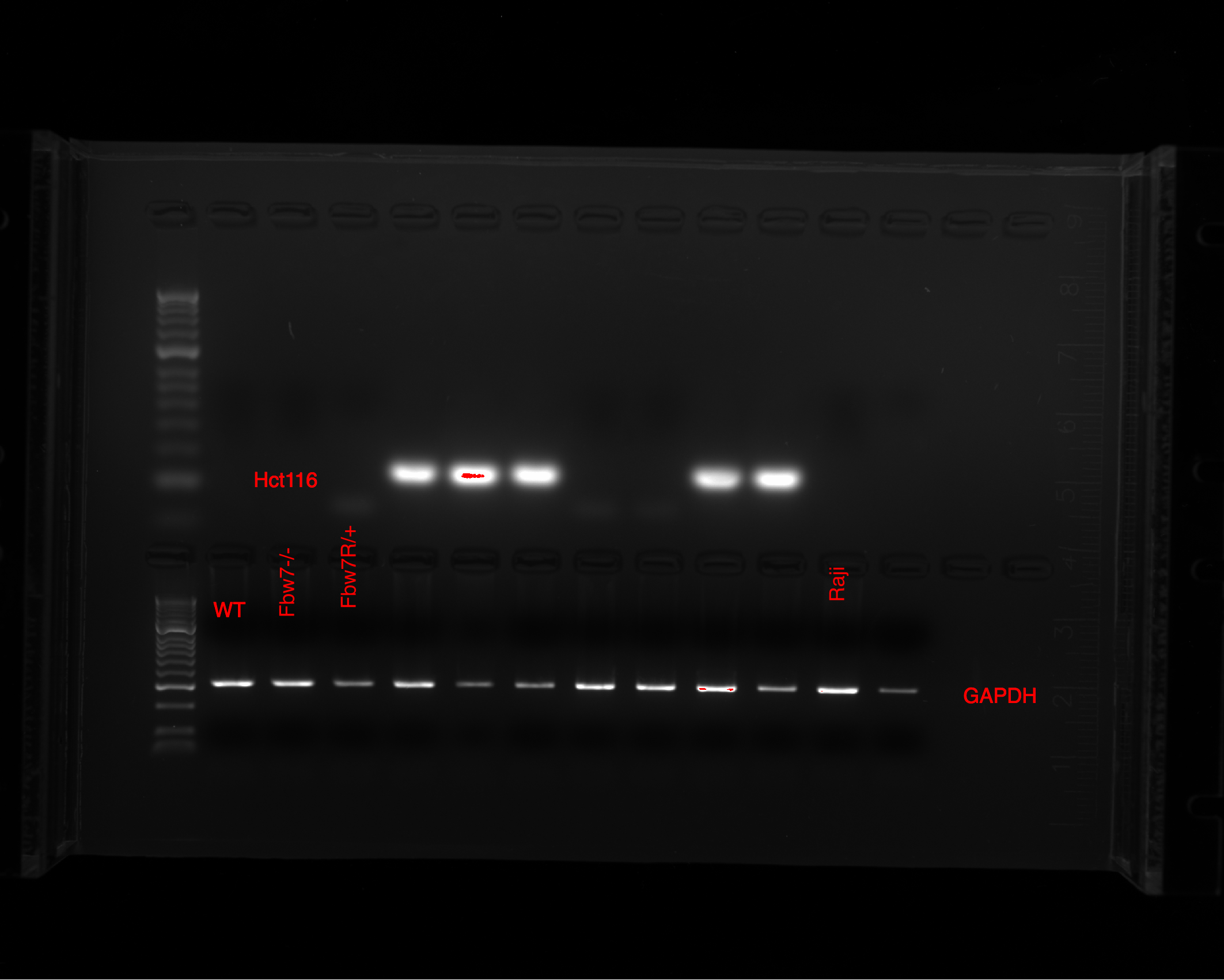

Supplement: Figure 5—source data 1. [file elife-74338-fig5-data1.zip › Figure 5- source data 1/Figure5C_labeled_GAPDH.tif]

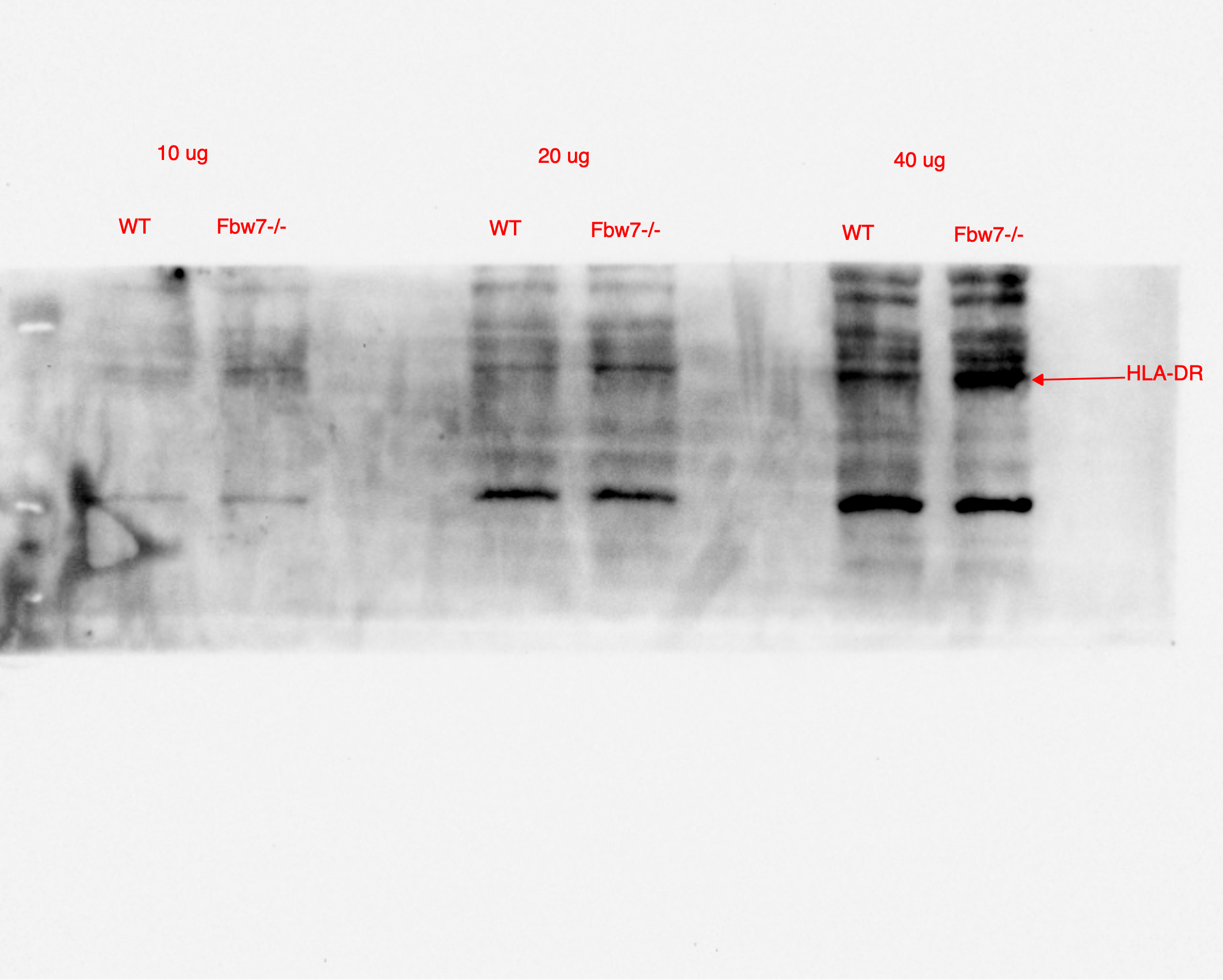

Supplement: Figure 5—figure supplement 2—source data 1. [file elife-74338-fig5-figsupp2-data1.zip › Figure 5 - source data 5/ Hct HLA 10h02m56s labeled Trial01.tif]

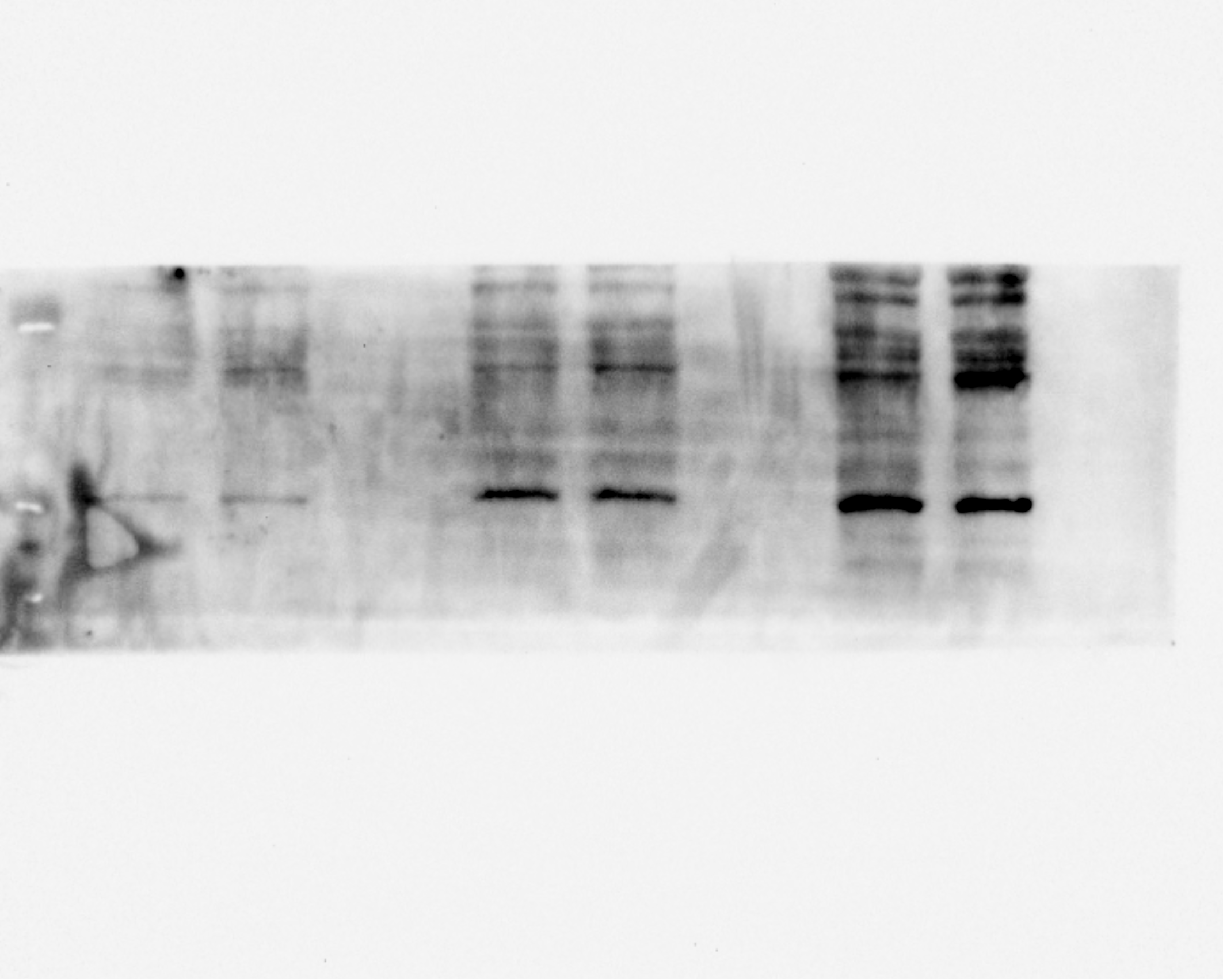

Supplement: Figure 5—figure supplement 2—source data 1. [file elife-74338-fig5-figsupp2-data1.zip › Figure 5 - source data 5/Hct HLA 10h02m56s Original Trial01.tif]

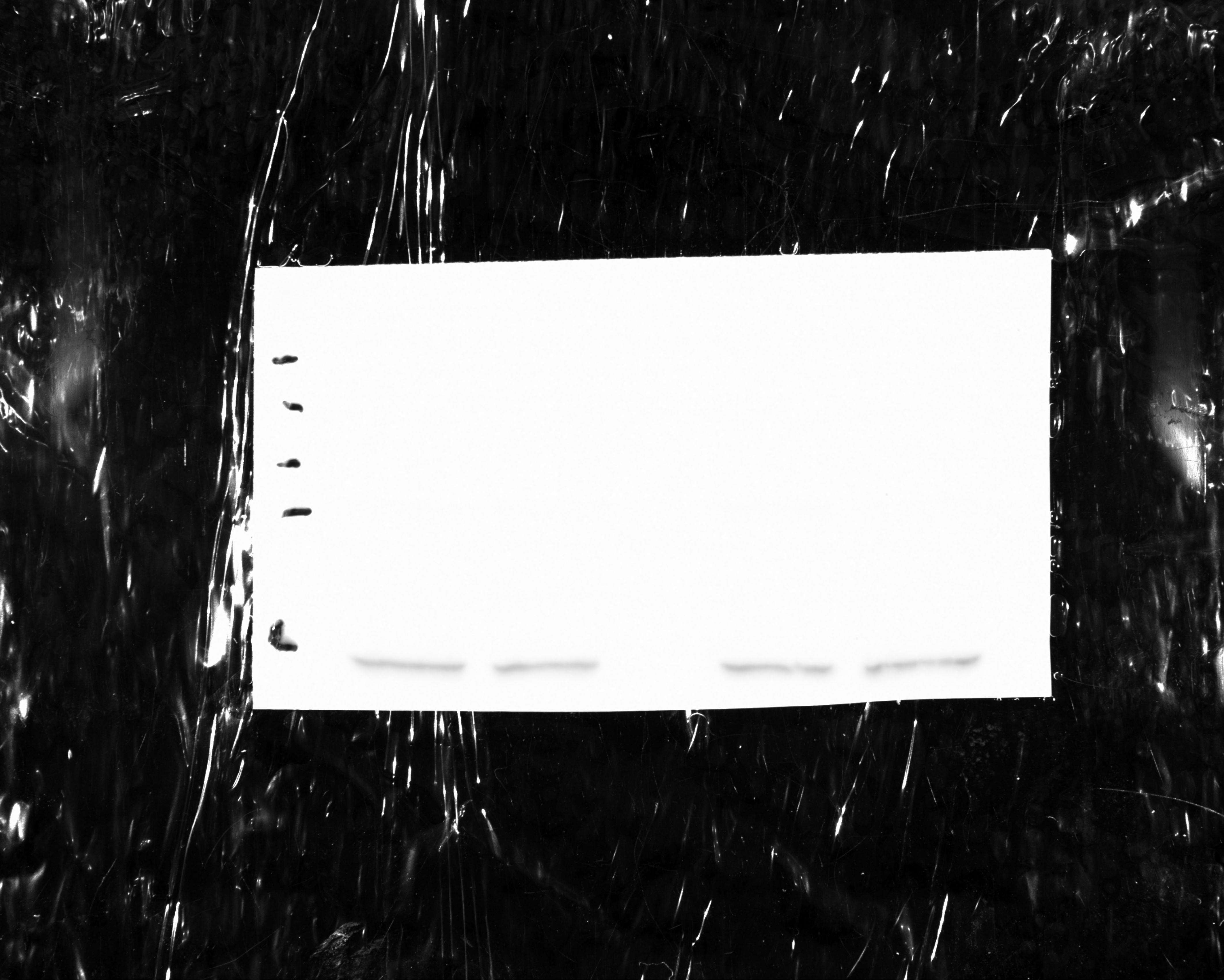

Supplement: Figure 5—figure supplement 2—source data 1. [file elife-74338-fig5-figsupp2-data1.zip › Figure 5 - source data 5/Tubulin Trial 02 original.tif]

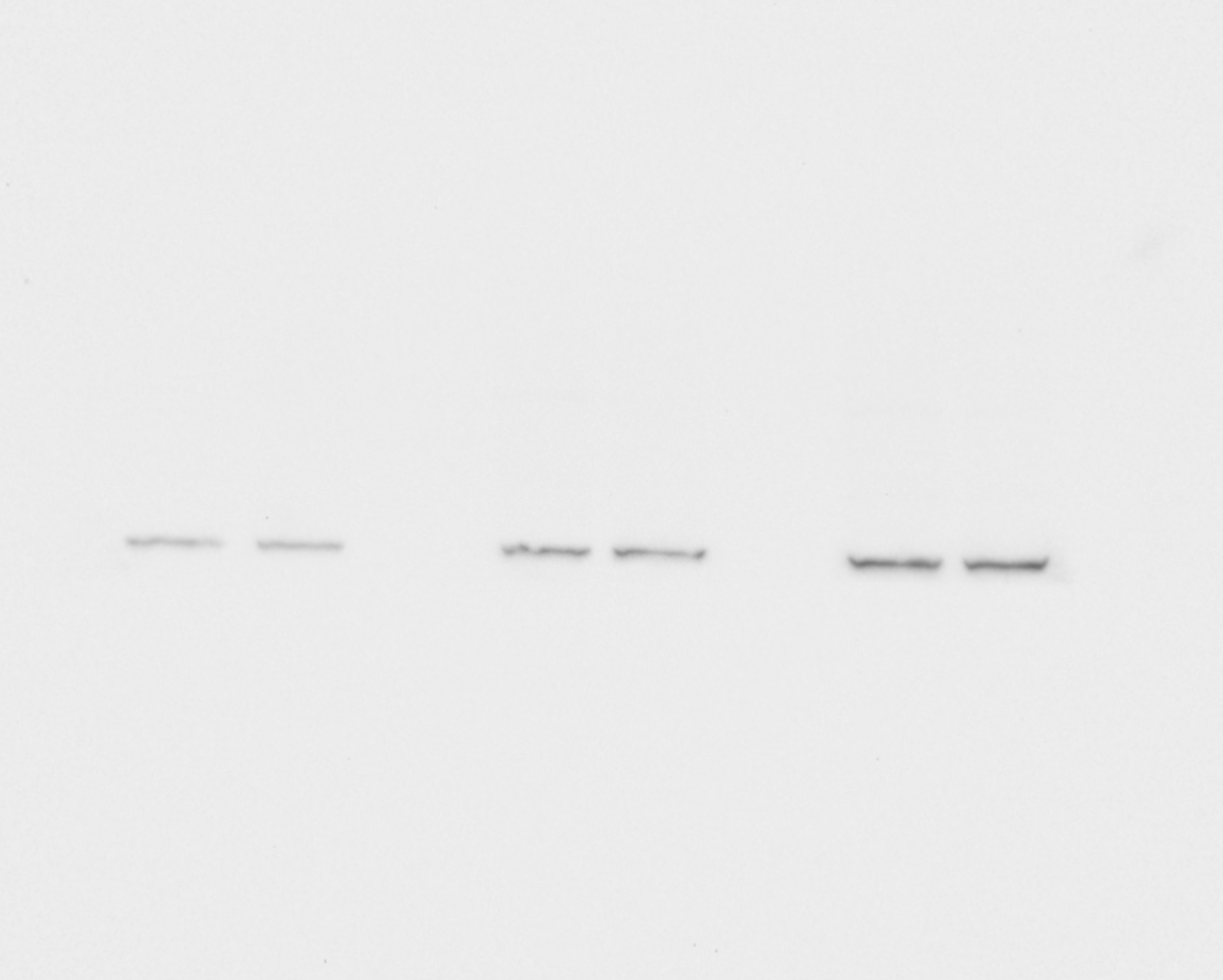

Supplement: Figure 5—figure supplement 2—source data 1. [file elife-74338-fig5-figsupp2-data1.zip › Figure 5 - source data 5/Tubulin Trial 01 original.tif]

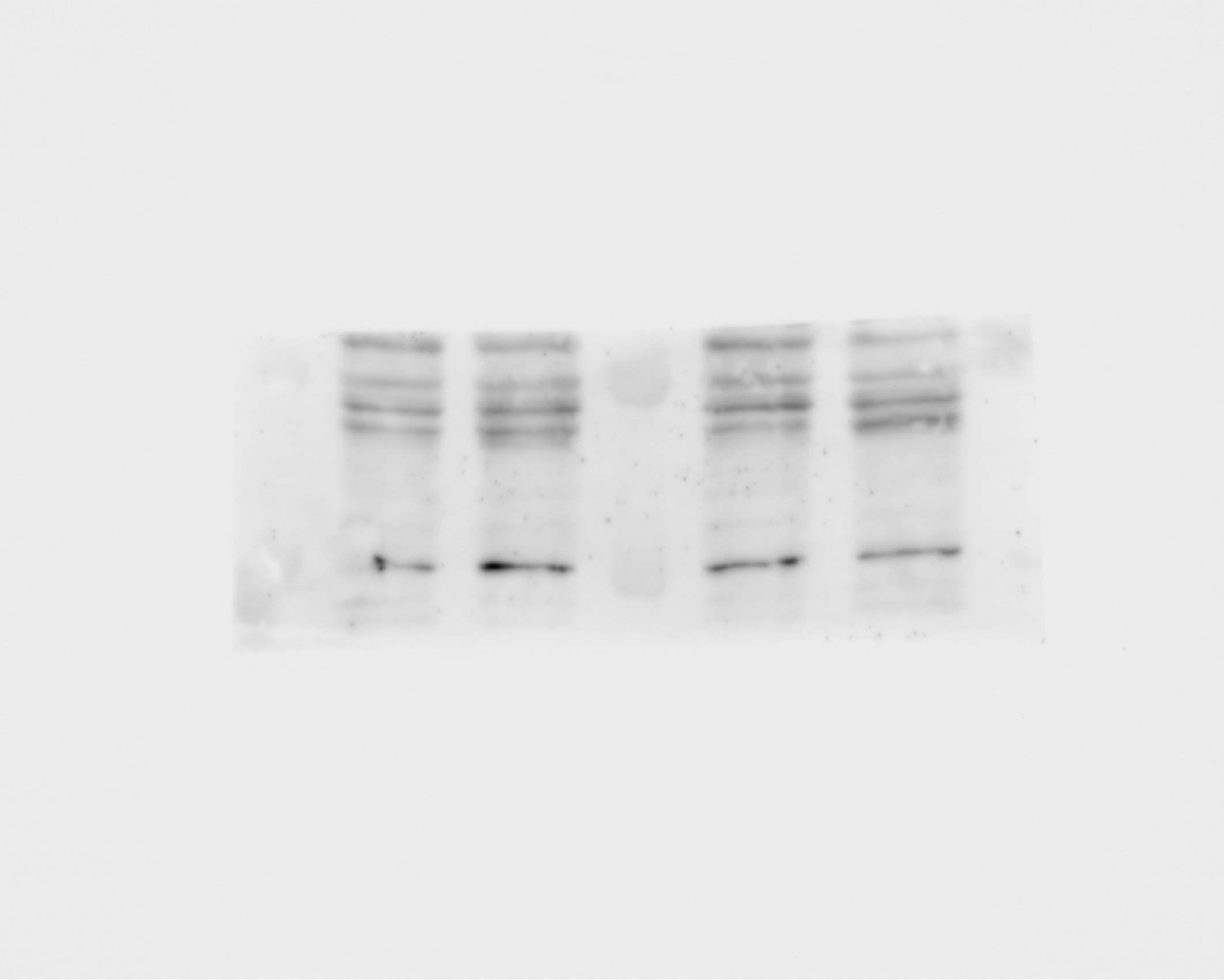

Supplement: Figure 5—figure supplement 2—source data 1. [file elife-74338-fig5-figsupp2-data1.zip › Figure 5 - source data 5/Hct HLA09h37m17s Original Trial02.tif]

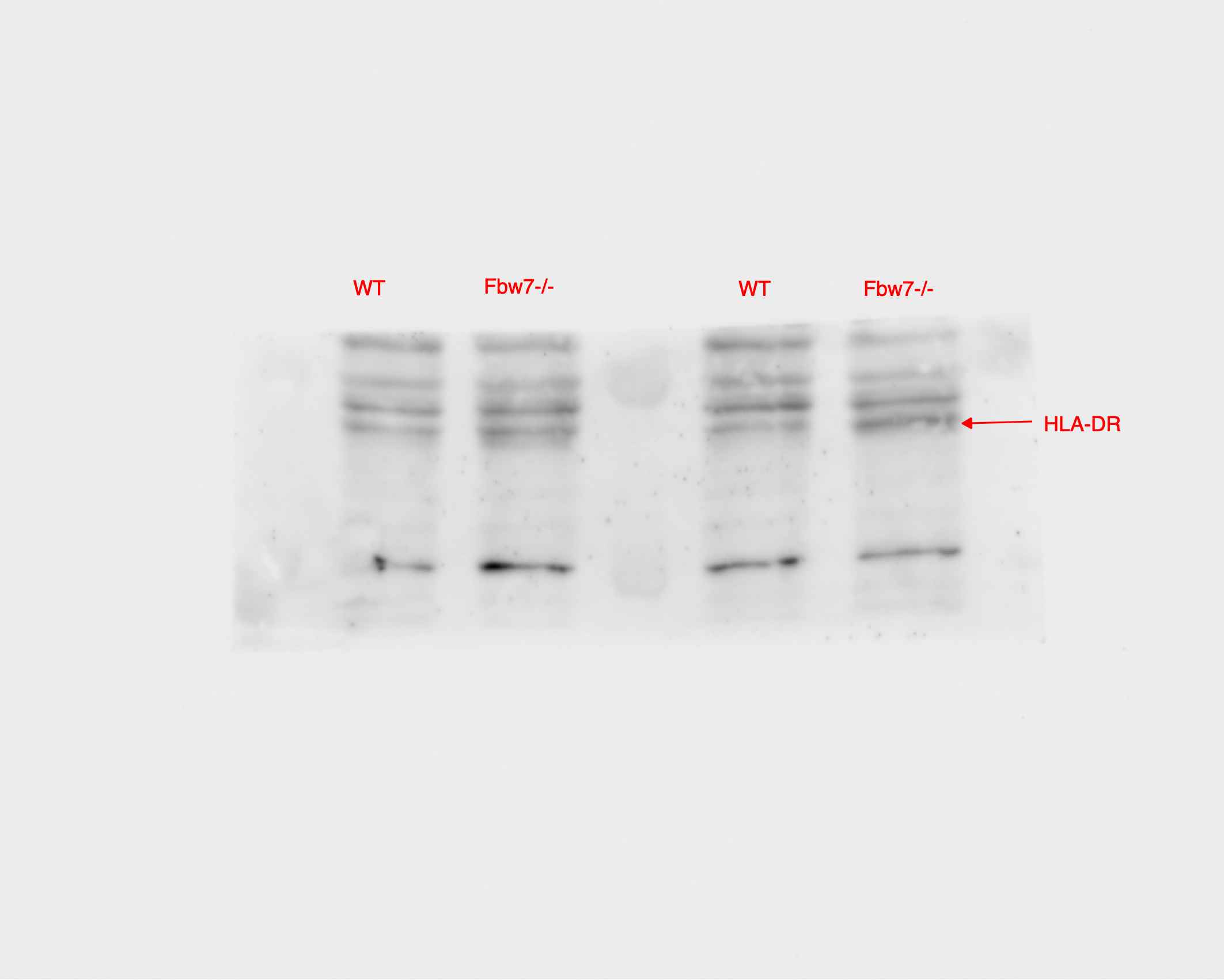

Supplement: Figure 5—figure supplement 2—source data 1. [file elife-74338-fig5-figsupp2-data1.zip › Figure 5 - source data 5/Hct HLA 09h37m17s labeled Trial 02.tif]

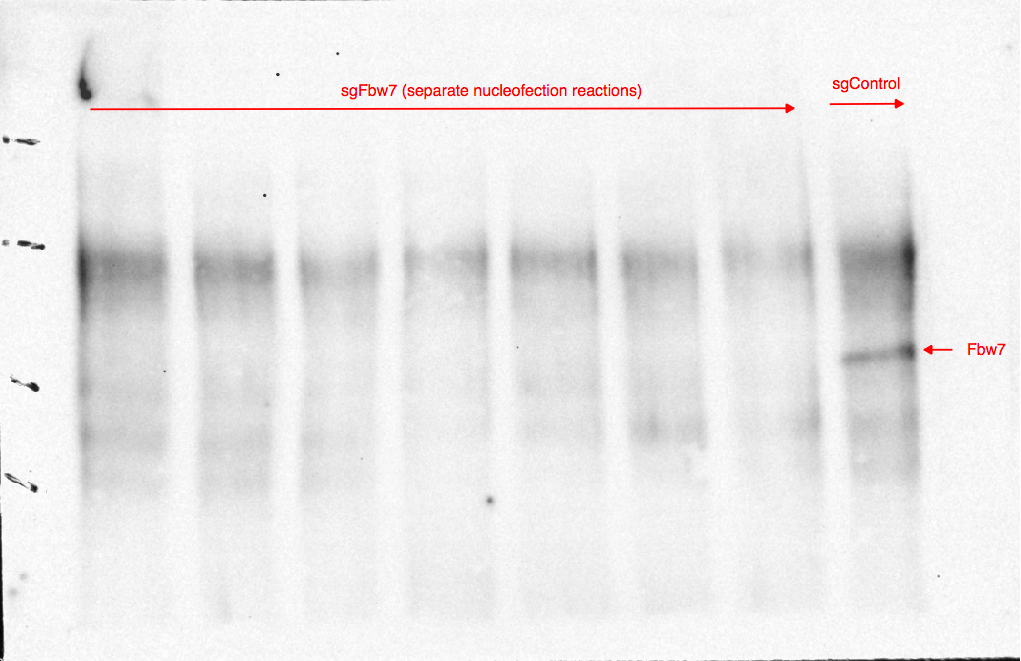

Supplement: Figure 6—figure supplement 1—source data 1. — This folder contains the original western blots for Figure 6—figure supplement 1A. Western blots that confirm the loss of Fbw7 in two others separately performed nucleofection reactions are also included. [file elife-74338-fig6-figsupp1-data1.zip › Figure 6 - source data 1/Figure6Supp1_KO-02_2019-01-25 labeled.tif]

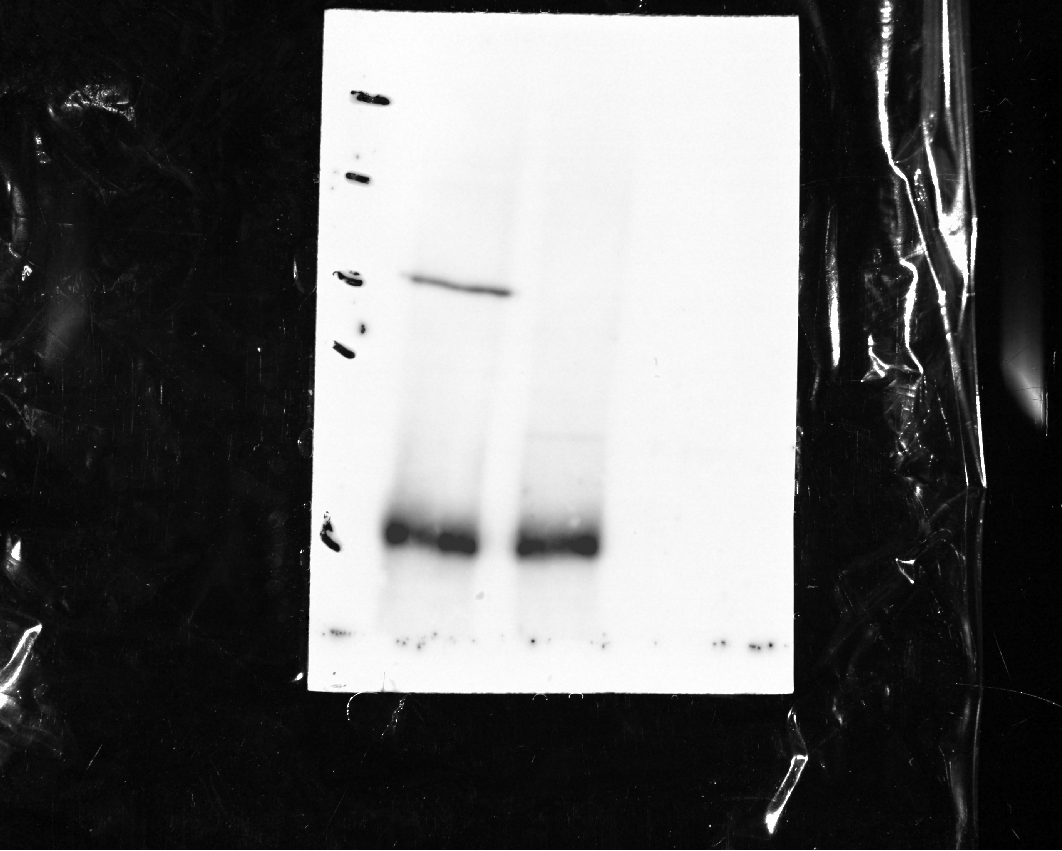

Supplement: Figure 6—figure supplement 1—source data 1. — This folder contains the original western blots for Figure 6—figure supplement 1A. Western blots that confirm the loss of Fbw7 in two others separately performed nucleofection reactions are also included. [file elife-74338-fig6-figsupp1-data1.zip › Figure 6 - source data 1/Figure6Supp1_KO-03.tif]

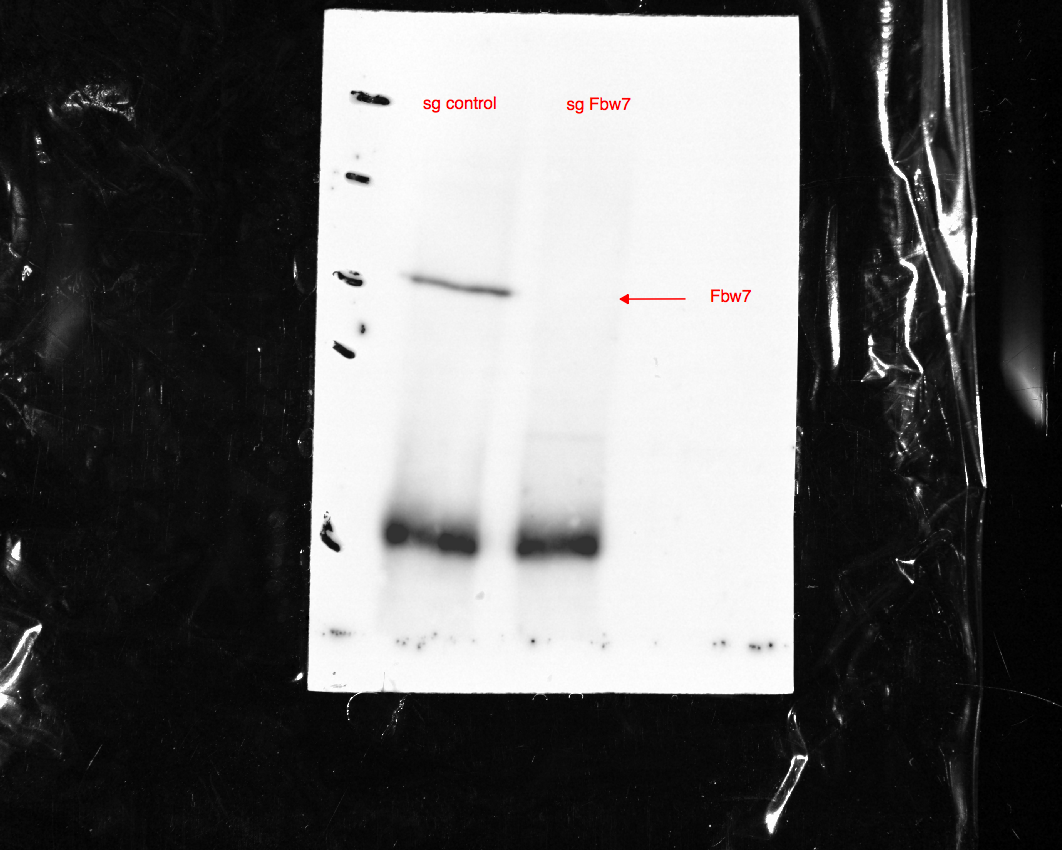

Supplement: Figure 6—figure supplement 1—source data 1. — This folder contains the original western blots for Figure 6—figure supplement 1A. Western blots that confirm the loss of Fbw7 in two others separately performed nucleofection reactions are also included. [file elife-74338-fig6-figsupp1-data1.zip › Figure 6 - source data 1/Figure6Supp1_KO-03_labeled.tif]

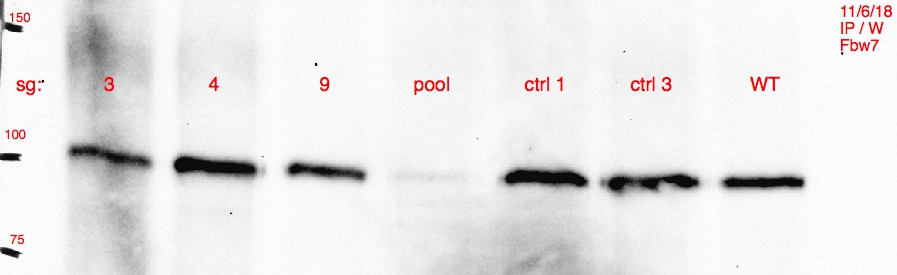

Supplement: Figure 6—figure supplement 1—source data 1. — This folder contains the original western blots for Figure 6—figure supplement 1A. Western blots that confirm the loss of Fbw7 in two others separately performed nucleofection reactions are also included. [file elife-74338-fig6-figsupp1-data1.zip › Figure 6 - source data 1/Figure6Supp1A_KO-01_labeled.jpg]

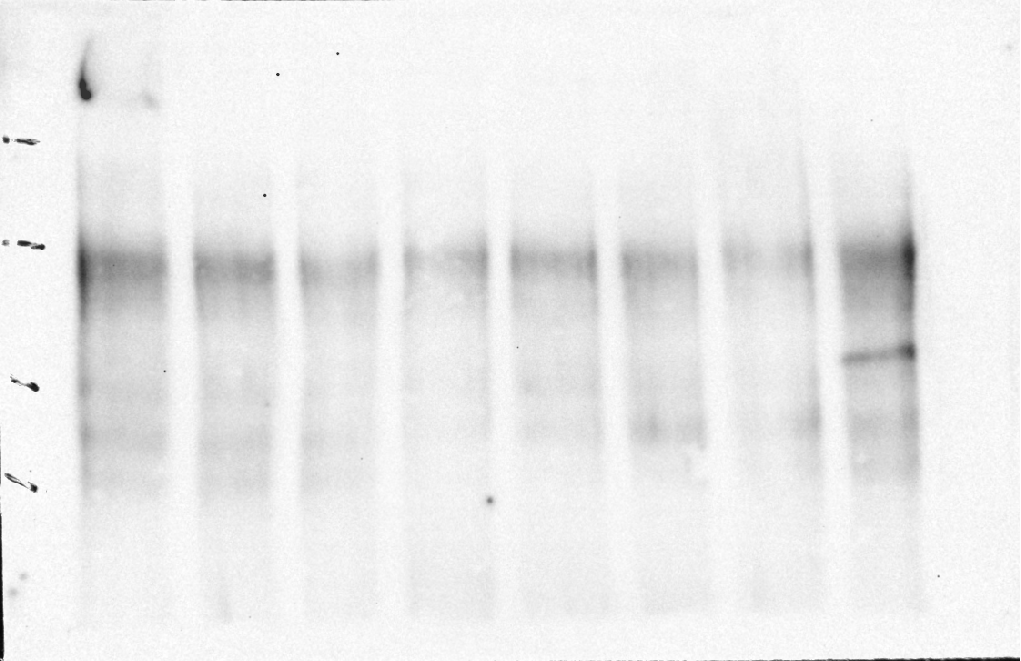

Supplement: Figure 6—figure supplement 1—source data 1. — This folder contains the original western blots for Figure 6—figure supplement 1A. Western blots that confirm the loss of Fbw7 in two others separately performed nucleofection reactions are also included. [file elife-74338-fig6-figsupp1-data1.zip › Figure 6 - source data 1/Figure6Supp1_KO-02_2019-01-25.tif]

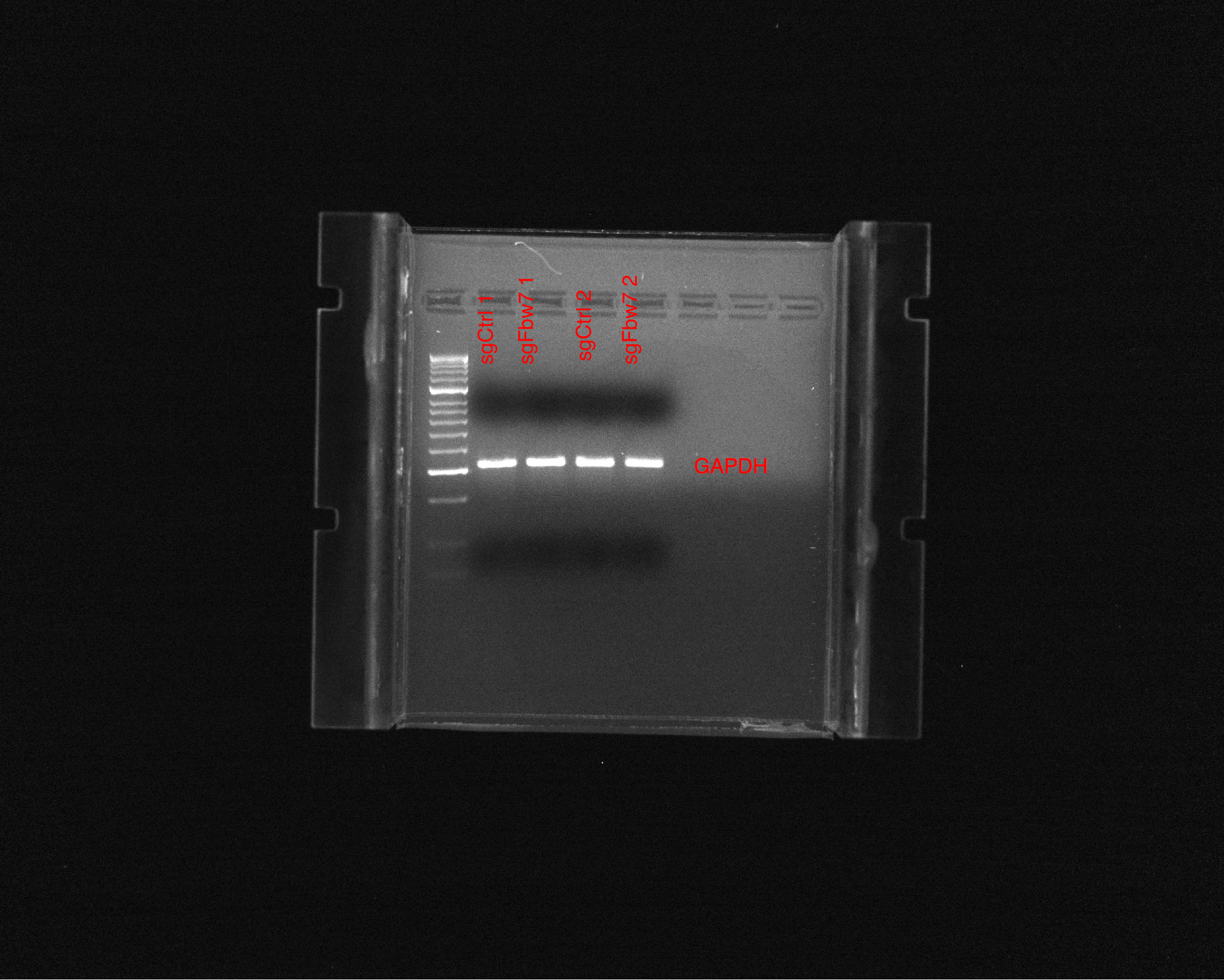

Supplement: Figure 6—figure supplement 3—source data 1. [file elife-74338-fig6-figsupp3-data1.zip › Figure 6 - source data 5/Nayanga 2021-09-16 12h32m25s labeled.tif]

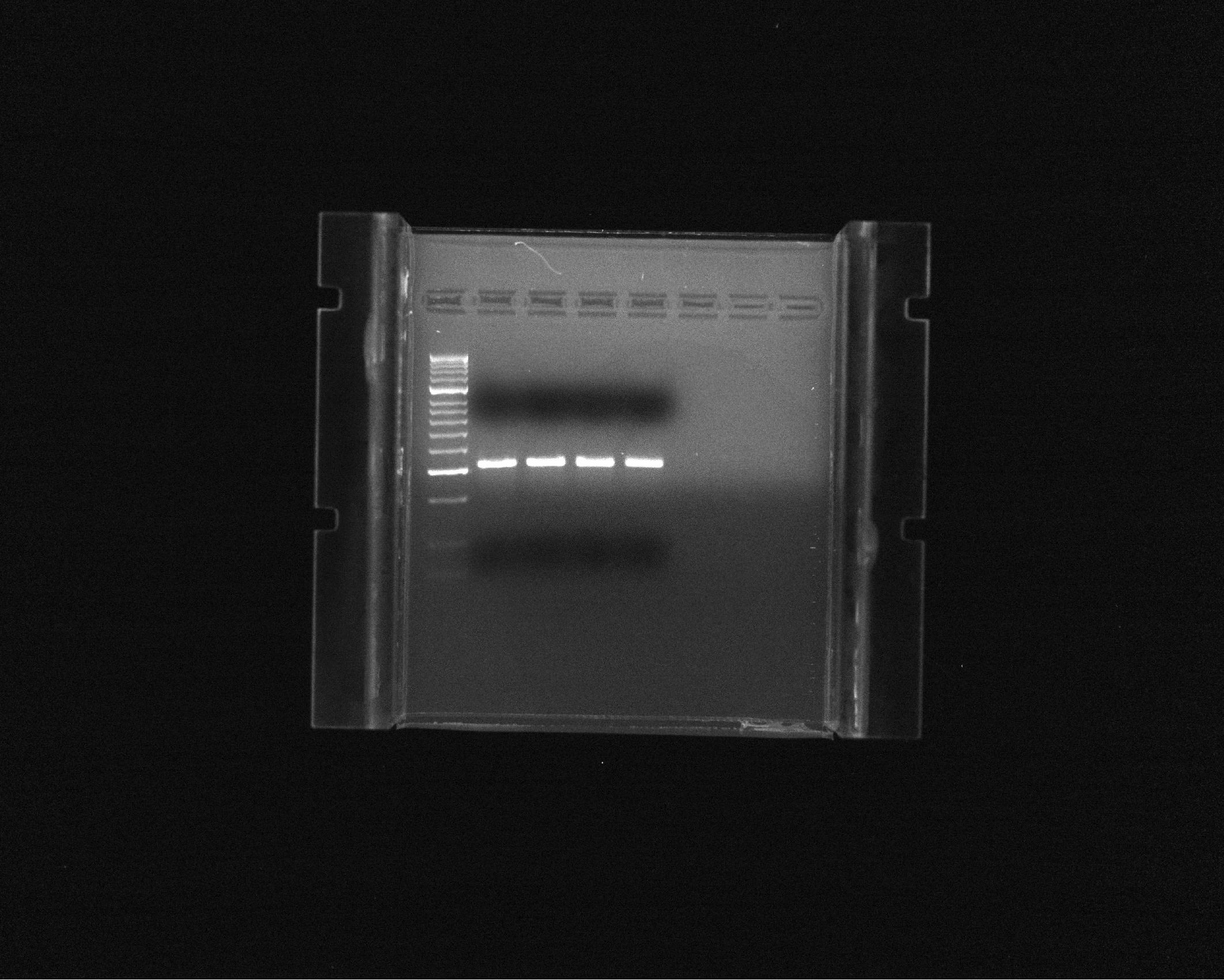

Supplement: Figure 6—figure supplement 3—source data 1. [file elife-74338-fig6-figsupp3-data1.zip › Figure 6 - source data 5/Nayanga 2021-09-16 12h32m25s(Ethidium Bromide).tiff]

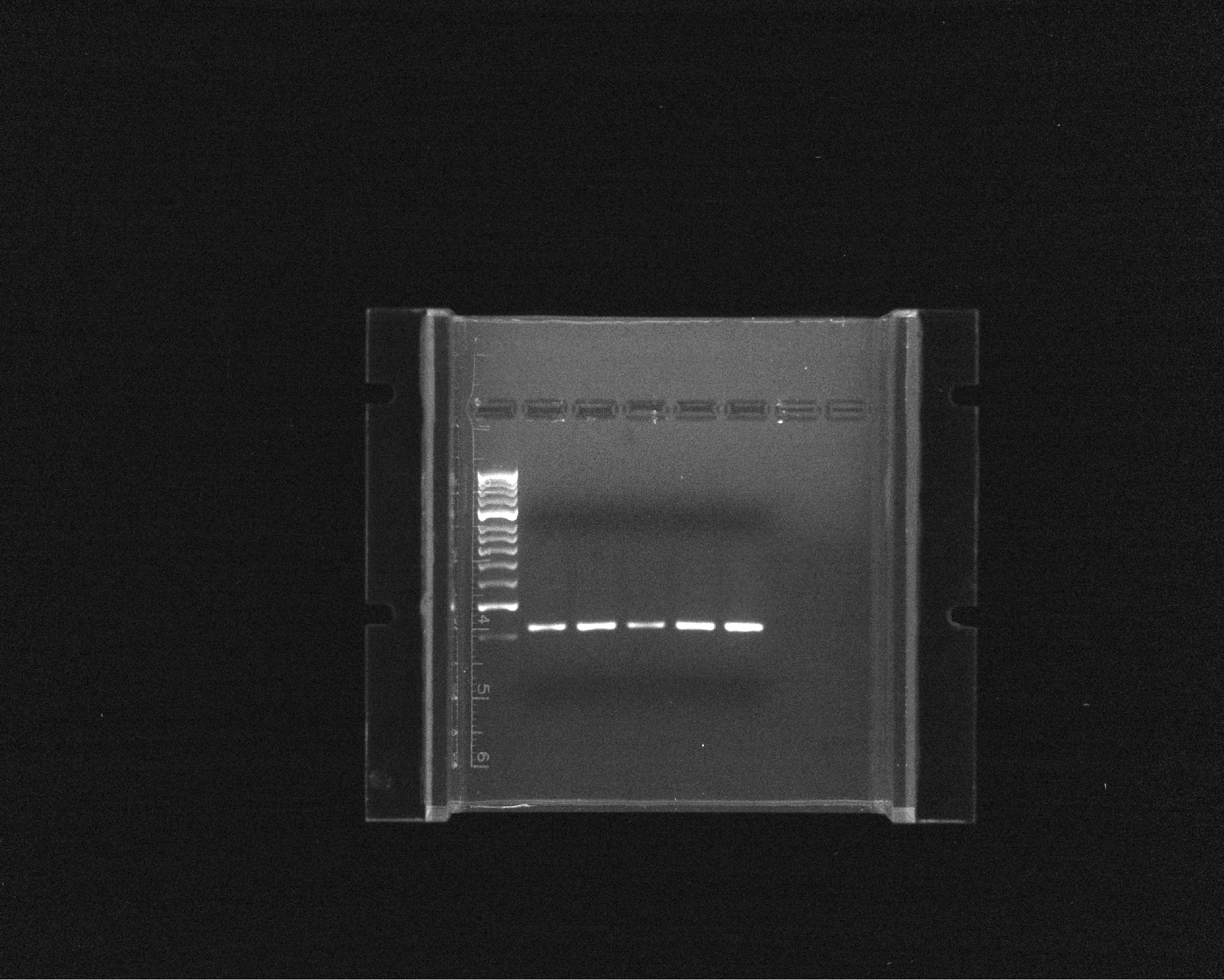

Supplement: Figure 6—figure supplement 3—source data 1. [file elife-74338-fig6-figsupp3-data1.zip › Figure 6 - source data 5/Nayanga 2021-08-05 12h56m59s(Ethidium Bromide).tiff]

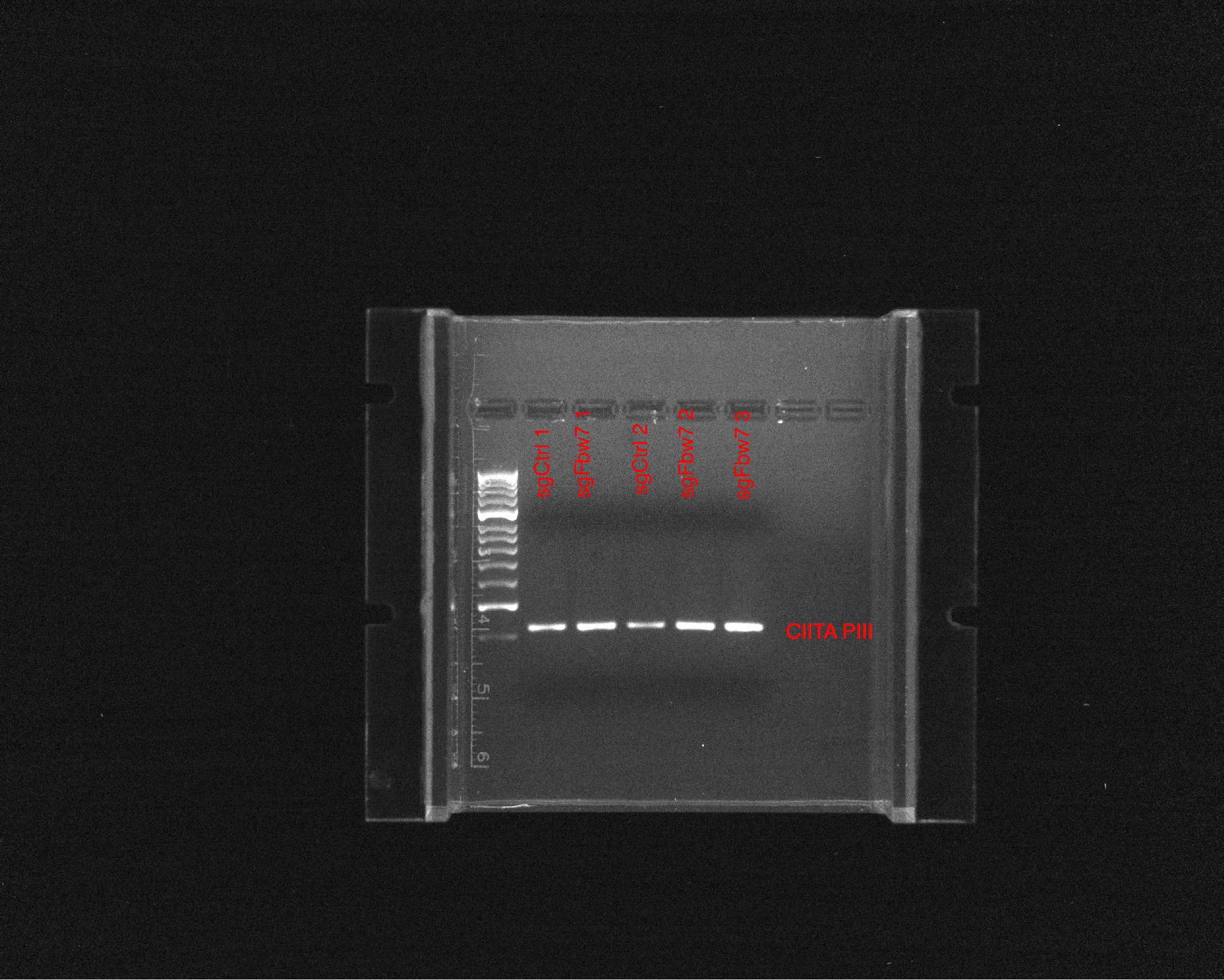

Supplement: Figure 6—figure supplement 3—source data 1. [file elife-74338-fig6-figsupp3-data1.zip › Figure 6 - source data 5/Nayanga 2021-08-05 12h56m59s labeled.tif]

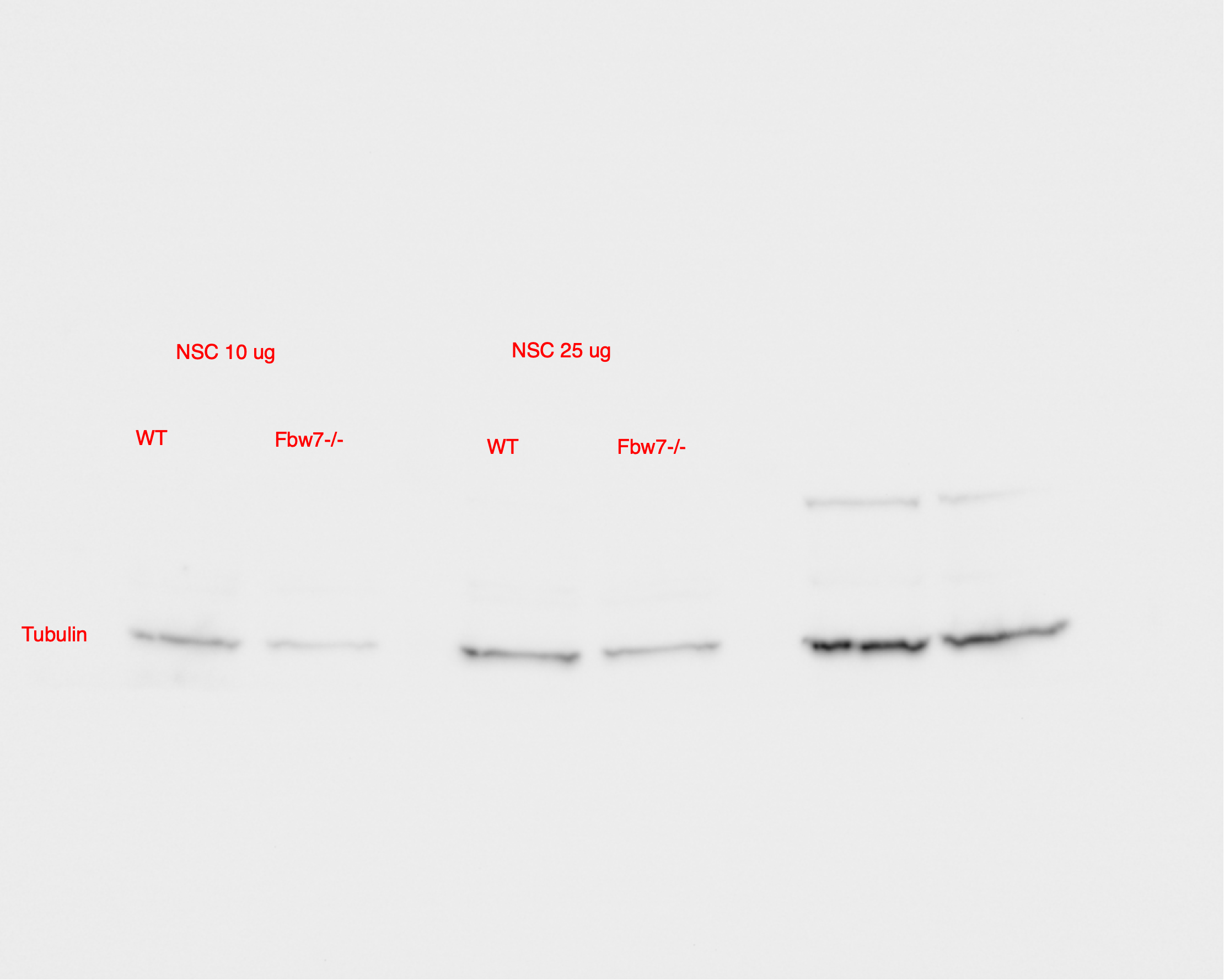

Supplement: Figure 6—figure supplement 5—source data 1. [file elife-74338-fig6-figsupp5-data1.zip › Figure 6 - source data 7/Tubulin NSC labeled.tif]

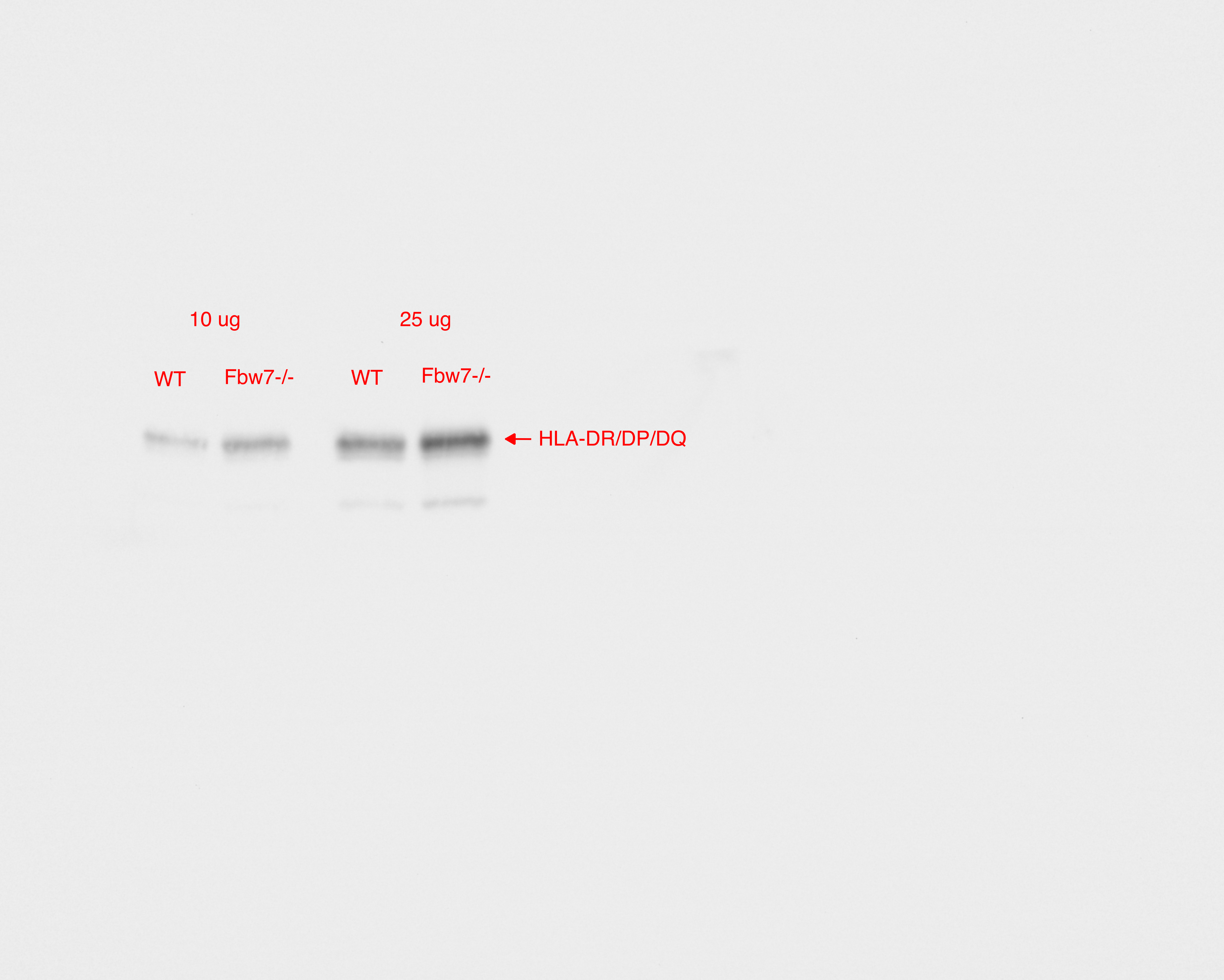

Supplement: Figure 6—figure supplement 5—source data 1. [file elife-74338-fig6-figsupp5-data1.zip › Figure 6 - source data 7/NSC HLADRPQ 11h38m36s Labeled.tif]

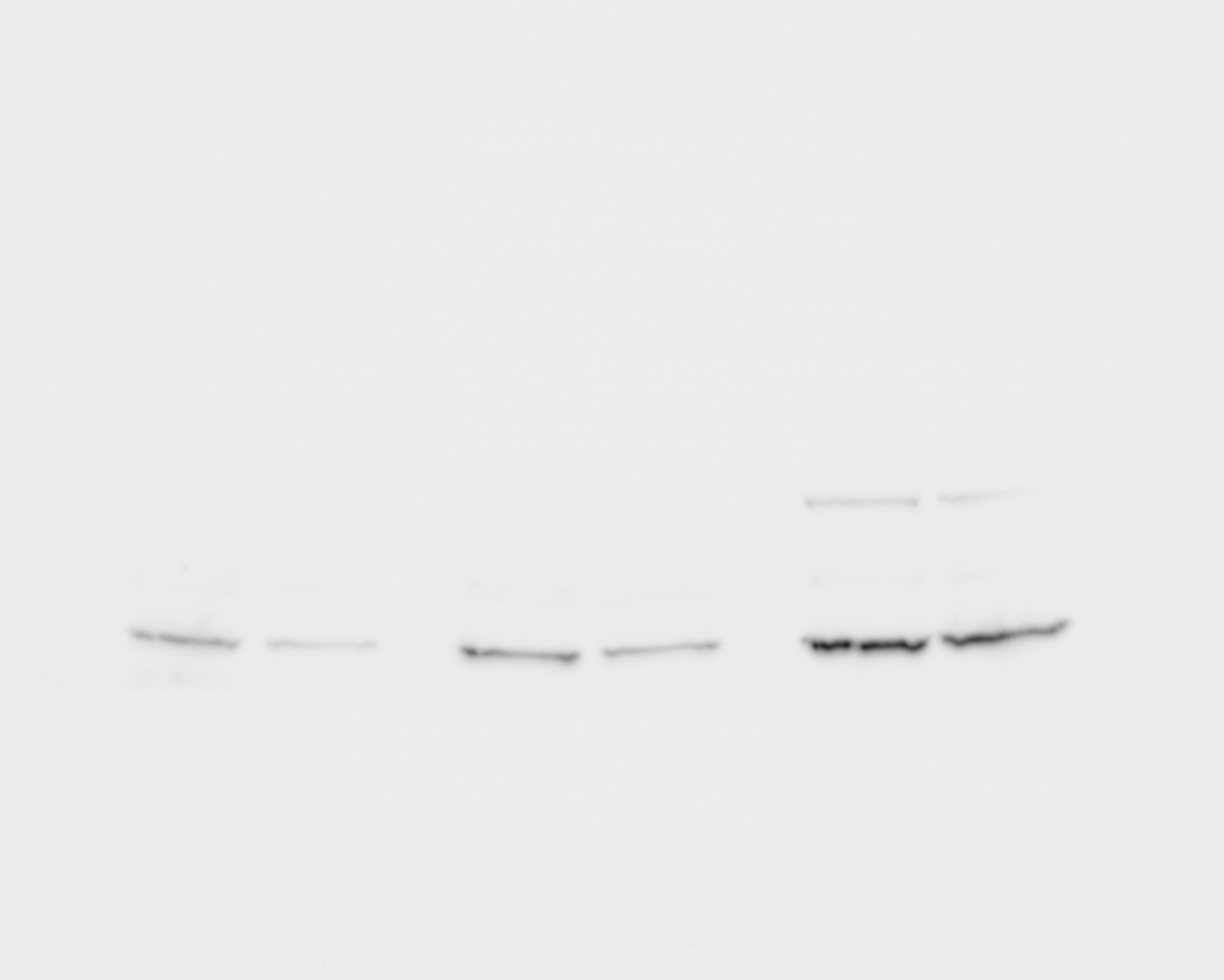

Supplement: Figure 6—figure supplement 5—source data 1. [file elife-74338-fig6-figsupp5-data1.zip › Figure 6 - source data 7/ Tubulin Original.tif]
